# Estimating the Burden of Sarcopenia in Adults With Type 2 Diabetes Mellitus: Implications for Metabolic Health

**DOI:** 10.7759/cureus.90161

**Published:** 2025-08-15

**Authors:** Deepika Paila, Ramesh Aggarwal, Anupam Prakash, Ghotekar L.H., Priya Bansal, Suman Gupta

**Affiliations:** 1 Internal Medicine, Lady Hardinge Medical College, New Delhi, IND

**Keywords:** asian working group 2019 for sarcopenia, bioimpedance analyzer, glycemic control, handgrip dynamometer, muscle mass, muscle strength, physical function, type 2 diabetes mellitus, sarcopenia

## Abstract

Background

Sarcopenia, the progressive loss of muscle mass and strength, is a significant complication in type 2 diabetes mellitus (T2DM) that worsens insulin resistance and glycemic control. Techniques such as a bioimpedance analyzer, handgrip strength (HGS) tests, and gait speed assessments serve to assess sarcopenia.

Aim

The aim of this study was to determine the proportion of T2DM patients with sarcopenia (T2DMS+) compared with non-diabetic patients and assess the association between sarcopenia and obesity in T2DM patients.

Materials and methods

For this observational cross-sectional study, all diabetic and non-diabetic male and female patients in the age group of 18-60 years were recruited during their routine outpatient visits. Sarcopenia was assessed using a bioimpedance analyzer for muscle mass, a handgrip dynamometer for muscle strength, and the Short Physical Performance Battery (SPPB) score for physical performance.

Observation and results

This study compared the prevalence and impact of sarcopenia in T2DM patients and non-diabetic patients. The study population included 100 T2DM patients and 100 controls. The mean age of the T2DM group (T2DMG) was 47.79 ± 8.64 years, slightly higher than that of the control group (CG), which was 44.31 ± 9.56 years. The gender distribution was consistent, with 70% (n = 70) of female patients and 30% (n = 30) of male patients in both groups. The body mass index (BMI) was similar between the T2DMG (25.9 ± 4.91 kg/m²) and the CG (26.76 ± 5.84 kg/m², p = 0.10). Hemoglobin A1c (HbA1c) levels were markedly higher in the T2DMG (8.05 ± 1.47%) compared with the CG (5.52 ± 0.65%, p < 0.05). Sarcopenia was significantly more prevalent in the T2DM group (53%, n = 53/100) than in the control group (17%, n = 17/100) with an odds ratio of 5.54 (95% confidence interval (CI): 2.84-10.81, p < 0.001), and more frequent in obese diabetics (64.15%, n = 34/53) compared to non-obese diabetics (35.85%, n = 19/53) with an odds ratio of 3.41 (95% CI: 1.38-8.39, p = 0.0001). Compared with the non-sarcopenic T2DM patients (47%, n = 47), sarcopenic T2DM patients (53%, n = 53) were older (52 ± 7.61 years and 43.02 ± 7.17 years, respectively, p < 0.05) and had longer diabetes duration (8.45 ± 5.45 years, p < 0.05). The sarcopenic T2DM patients also had less appendicular lean mass (ALM) (18.5 ± 4.14 kg compared with 21.19 ± 3.95 kg, p < 0.0014), reduced HGS (14.97 ± 3.83 kg compared with 25.78 ± 4.34 kg, p < 0.05), lower SPPB scores (6.49 ± 1.4 compared with 10.06 ± 0.76, p < 0.05), and higher HbA1c (8.54 ± 1% compared with 7.5 ± 1.47%, p = 0.00037).

Conclusion

T2DM patients exhibited lower appendicular lean mass, muscle strength, and physical performance, and had significantly lower handgrip strength and SPPB scores than the non-sarcopenic T2DM patients. Further, sarcopenia was more pronounced in the older patients and those with a longer duration of diabetes.

## Introduction

The global burden of type 2 diabetes mellitus (T2DM) is increasing rapidly, primarily because of sedentary lifestyles, unhealthy dietary habits, and an aging population. T2DM is now increasingly linked to sarcopenia, a condition characterized by the progressive loss of muscle mass, strength, and function [1,2]. Although previously regarded as an age-related condition, sarcopenia is now frequently identified in younger adults with T2DM. Sravya et al. observed that up to 22% of T2DM patients may have sarcopenia, a proportion significantly higher than in the general population [1]. This finding has raised concern about associated risks such as falls, fractures, disability, and mortality.

T2DM is fundamentally driven by insulin resistance, which not only impairs glucose uptake but also disrupts muscle protein synthesis [3]. Over time, the impairment and disruption lead to progressive muscle loss, contributing to sarcopenia. In parallel, insulin resistance promotes weight gain and visceral fat accumulation, which, in turn, triggers chronic low-grade inflammation [4]. This pro-inflammatory state exacerbates muscle degradation, thus reinforcing the bidirectional links among diabetes, obesity, and sarcopenia.

A particularly concerning condition is sarcopenic obesity, in which excess fat mass coexists with reduced muscle mass. This phenotype is common in individuals with T2DM and is associated with worsened metabolic control and physical function. Excess fat mass accelerates muscle breakdown and increases insulin resistance, while reduced muscle mass impairs glucose disposal, further diminishing glycemic control [5]. In addition, hormonal imbalances (e.g., reduced testosterone and growth hormone levels) and mitochondrial dysfunction contribute to muscle deterioration.

Sarcopenia in T2DM is strongly associated with increased risk of cardiovascular disease, disability, hospitalization, and premature death. Despite its serious implications, sarcopenia often remains underdiagnosed, particularly in its early stages. Therefore, early detection and intervention are essential for improving clinical outcomes and preserving quality of life in patients with T2DM. The aim of this study was to determine the proportion of T2DM patients with sarcopenia (T2DMS+), to assess how this proportion differs from that in non-diabetic patients, and to evaluate the distribution of sarcopenia among obese and non-obese individuals within the diabetic group.

## Materials and methods

Study sample and data collection

For this observational cross-sectional study, we evaluated 100 patients with T2DM (T2DM group (T2DMG)) recruited during routine outpatient visits to a tertiary care hospital between May 2023 and October 2024. We included patients in the age group of 18-60 years with known or newly diagnosed T2DM as defined by the 2023 American Diabetes Association guidelines [6]. The lower age limit of 18 years was chosen to allow early detection of sarcopenia in younger adults with T2DM, given emerging evidence that metabolic alterations in diabetes can precipitate muscle decline earlier in life. We excluded patients with severe comorbidities such as chronic kidney disease, chronic liver disease, cardiovascular disease, systemic infection, thyroid disease, and malignancy. We also excluded patients who were using drugs that can modify body composition, such as corticosteroids, antithyroid drugs, and oral contraceptives, patients who were taking hormonal or nutritional supplementation, professional athletes, bedbound patients, moribund patients, and patients who could not follow the evaluation protocol.

The control group (CG) consisted of 100 non-diabetic subjects in the same age group (i.e., 18-60 years). Individuals in this group were matched by age and sex with the individuals in the T2DM group. The members of the CG underwent the same evaluations as the members of the T2DMG.

Anthropometric measures

Body weight was measured with the participants wearing light clothes and no shoes, height was measured using a measuring tape, and the participants’ body mass index (BMI) was calculated accordingly. Waist circumference was measured at the midpoint between the lower costal margin and the iliac crest. Values of ≥90 cm and ≥80 cm in men and women, respectively, were considered obese [7]. Sarcopenia was assessed using the following three techniques.

Muscle Mass Measurement

A bioimpedance analyzer is a medical device used to measure the impedance or resistance to the flow of electrical current through the body. The device commonly serves to assess body composition, particularly the ratio of fat to lean body mass [8]. Appendicular lean mass (ALM) measured by a bioimpedance analyzer is calculated as the sum of lean mass values obtained in the arms and legs. ALM is used to calculate the appendicular lean mass index (ALMI), which is ALM/height². The cutoff values used for defining sarcopenia based on the ALMI were <7 kg/m² in men and <5.7 kg/m² in women [9].

Handgrip Strength (HGS) Measurement

We measured the participants’ HGS using a medical hand grip dynamometer (Camry electronic hand dynamometer model EH101). This device measures HGS with a pressure-sensitive sensor. When a person squeezes the handles, the device records the maximum force exerted in kilograms or pounds. The device is commonly used to assess muscle strength, rehabilitation progress, and general fitness levels. To use it, the patients hold the device in one hand and squeeze as hard as possible while keeping their arm at a right angle. The device then displays the grip strength digitally. It is easy to operate, and the results can provide insight into overall muscle health and function. It records the mean value (in kilograms) of three consecutive measurements of the dominant arm gripping a dynamometer. Low muscle strength was described as <28 kg in men and <18 kg in women according to the cutoff points for the Asian population [9].

Physical Performance Assessment

The Short Physical Performance Battery (SPPB) score is used to assess physical performance. It consists of a balance test, a gait speed test, and a chair stand test. The maximum score calculated from the three tests is 12, and a score ≤ 9 is considered to indicate low physical performance [9].

Sarcopenia diagnosis

**Figure 1:**
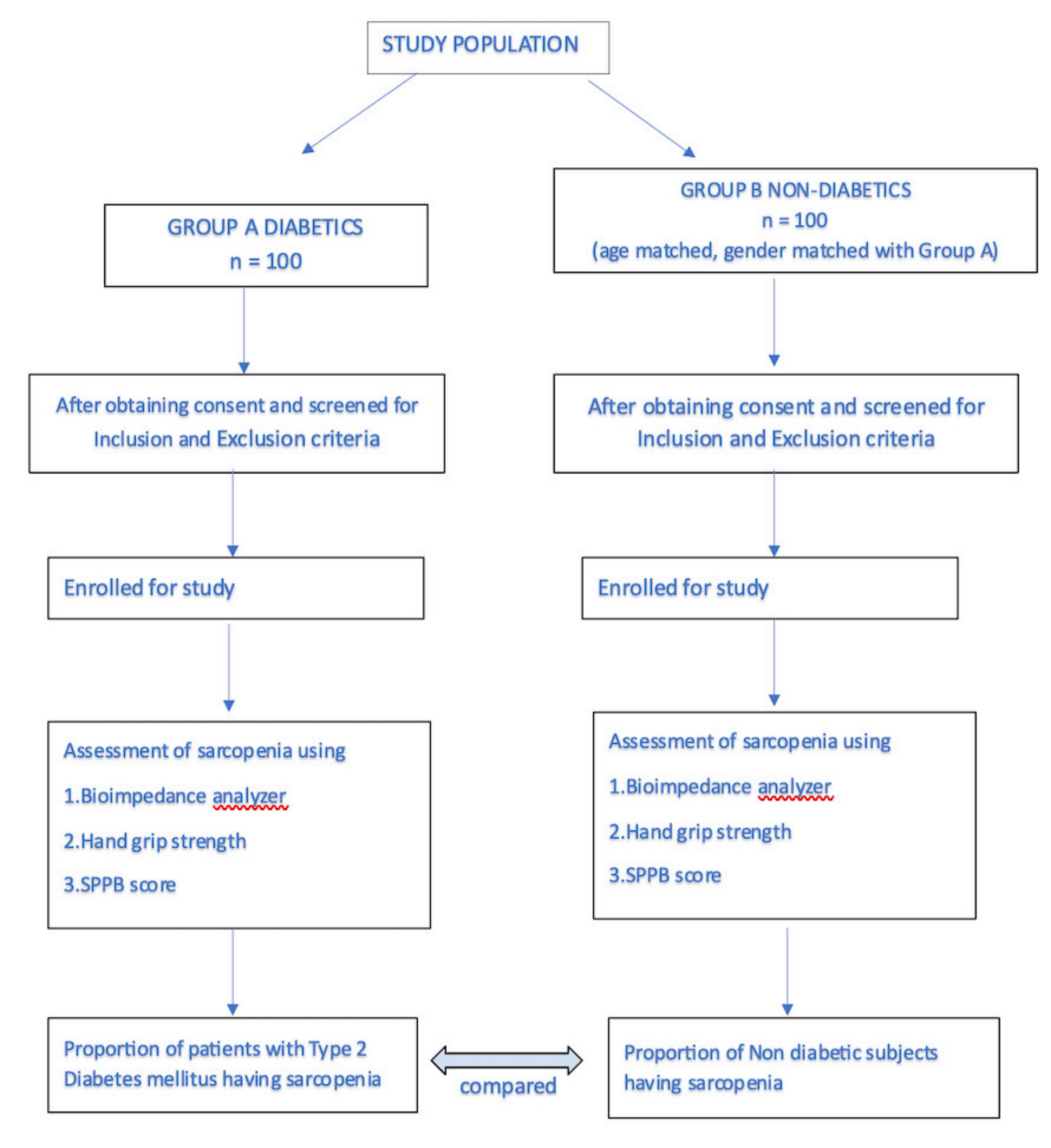
Flowchart showing methodological recruitment of study population and allocation to subgroups n: total number of patients, SPPB: Short Physical Performance Battery

The Asian Working Group for Sarcopenia recommends measuring muscle strength and physical performance as the screening test. Subjects with low muscle strength and low physical performance should be labeled as possibly having sarcopenia [9]. In clinical practice, this label is sufficient to trigger assessment of causes and start intervention. These subjects should be further evaluated by measuring their muscle mass for the diagnosis of sarcopenia. The subjects are labeled “diagnosed sarcopenia” when low muscle mass is present in addition to low muscle strength and/or reduced physical performance. In this study, patients who were labeled as having possible or diagnosed sarcopenia were assigned to the sarcopenic category. For the purpose of this study, participants meeting criteria for either “possible” or “diagnosed” sarcopenia (per Asian Working Group for Sarcopenia 2019) were grouped together, as both categories are considered clinically relevant and warrant intervention; this approach allowed us to estimate the overall burden of sarcopenia in T2DM more comprehensively. Figure 1 presents a flowchart depicting the recruitment and allocation methods.

**Figure 2:**
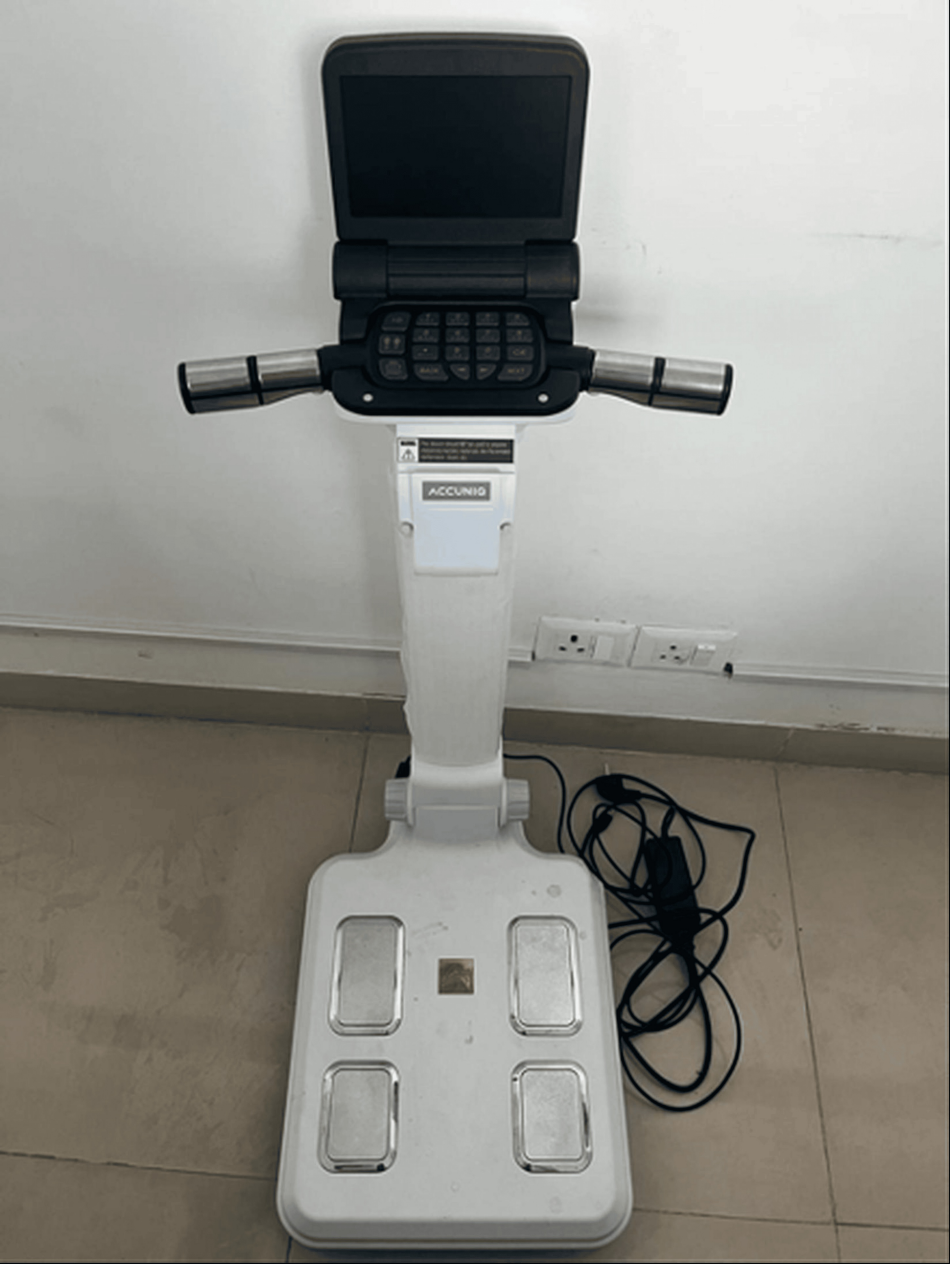
ACCUNIQ BC300 bioimpedance analyzer The ACCUNIQ BC300 works on the principle of bioimpedance analysis (i.e., electric current passes through the body at a differential rate depending on body composition). The patient stands on the metal plates on the base, which act as an electrode, and then grasps the horizontal bar, which also has circumferential electrodes. The machine generates an electric current between the two electrodes that passes through the body and then measures the drop in voltage, which is a measure of the resistance provided by individual tissues and, hence, the physical composition. To ensure accurate results, patients are instructed to avoid eating, drinking, or exercising for at least 2-3 hours before the test, remove any metal accessories, and stand barefoot on the footpads. Contraindications include pregnant women, individuals with serious heart conditions, and individuals with implanted electronic devices such as pacemakers, for the electrical current may interfere with these devices. The image of the bioimpedance analysis machine was captured by the authors.

The bioimpedance analyzer (ACCUNIQ BC300, SELVAS Healthcare, Daejeon, South Korea) used in this study is shown in Figure 2.

Statistical analysis

Data were entered into a Microsoft Excel spreadsheet (Microsoft Corp., Redmond, WA), and analysis was conducted using Statistical Package for the Social Sciences (SPSS) version 25.0 (IBM Corp., Armonk, NY). Continuous variables were represented as mean ± standard deviation (SD) or median with interquartile range. Categorical variables were represented as numbers and percentages (%). The Kolmogorov-Smirnov test served to assess the variables for normality, and Q-Q plots, visual inspection of the histograms, and z-scores served to assess the degree of skewness and kurtosis. All tests of significance were two-tailed, and statistical significance was defined as p < 0.05. In comparing the values of a variable between two groups at a particular point in time, the Mann-Whitney U Test was used for non-parametric variables, and Student’s t-test was used for parametric variables. The Chi-square test was used to compare the difference in the proportions between the two groups. Appropriate graphs, such as pie charts, bar diagrams, and histograms, have been constructed.

## Results

The study population consisted of 100 T2DM subjects and 100 non-diabetic subjects. The average age of the subjects in the T2DMG was 47.79 ± 8.64 years, and that of the non-diabetic subject group was 44.31 ± 9.56 years. The female:male ratio in both the T2DMG and the CG was 70/100 (70%) female patients and 30/100 (30%) male patients. The mean duration of diabetes was 6.249 ± 5.25 years, and the mean hemoglobin A1c (HbA1c) level was 8.05 ± 1.47%. Table 1 shows the baseline characteristics of the study population.

**Table 1:** Baseline characteristics of the study population Data presented as mean ± standard deviation ^#^Independent t-test *Significant difference BMI: body mass index, WC: waist circumference, HbA1c: hemoglobin A1c, T2DM: type 2 diabetes mellitus, SD: standard deviation

Parameter	T2DM group	Control group	p-value^#^
Age (years ± SD)	47.79 ± 8.64	44.31 ± 9.56	0.0075*
Gender (female:male)	70:30	70:30	-
BMI (kg/m^2^)	25.9 ± 4.91	26.76 ± 5.84	0.10
WC (cm)	84.43 ± 9.65	87.6 ± 11.58	0.03
HbA1c	8.05 ± 1.47	5.52 ± 0.65	<0.05*

Table 2 shows the proportion of T2DM patients with sarcopenia.

**Table 2:** Proportion of T2DM patients with sarcopenia N: total number of patients, T2DM: type 2 diabetes mellitus

	T2DM patients (N = 100)
With sarcopenia	53 (53%)
Without sarcopenia	47 (47%)

Among the 100 T2DM patients included in the study, 53 (53%) were identified as having sarcopenia, while the remaining 47 (47%) did not have sarcopenia. This finding highlights the high prevalence of sarcopenia in the diabetic population studied. Table 3 shows the proportion of patients with sarcopenia in the T2DM group by age group.

**Table 3:** Proportion of T2DM patients with sarcopenia by age group n: number of patients, T2DM: type 2 diabetes mellitus

Age group	T2DM patients with sarcopenia (n = 53)
18-32 years	2/53 (3.77%)
33-47 years	11/53 (20.75%)
48-60 years	40/53 (75.47%)
Total	53/53 (100%)

When stratified by age, the majority of the T2DM patients with sarcopenia belonged to the 48-60 years age group, accounting for 75.47% (40/53) of the cases. Smaller proportions were observed in the 33-47 years group (20.75%, 11/53) and the 18-32 years group (3.77%, 2/53). These results indicate an age-related increase in the prevalence of sarcopenia among diabetic patients. Table 4 depicts the proportion of non-diabetic subjects with sarcopenia, which was found to be 17/100 (17%).

**Table 4:** Proportion of non-diabetic patients with sarcopenia by age group n: number of patients

Age group	Non-diabetic subjects with sarcopenia (n = 17)
18-32 years	0 (0%)
33-47 years	0 (0%)
48-60 years	17/100 (17%)

The analysis presented in Table 3 and Table 4 shows that, in the non-diabetic population, sarcopenia was present only in the 48-60 years age group (17/100, 17%) while, in the diabetic population, sarcopenia was present in the 18-32 years (2/53, 3.77%) and 33-47 years (11/53, 20.75%) groups and was most prevalent in the 48-60 years group (40/53, 75.47%). The proportion of T2DM patients with sarcopenia was 53/100 (53%), compared with 17/100 (17%) in the non-diabetic patients, with a p-value of <0.05 using the Chi-square test. Sarcopenia was significantly more prevalent in the T2DM group (53%, n = 53/100) than in the control group (17%, n = 17/100) with an odds ratio of 5.54 (95% confidence interval (CI): 2.84-10.81, p < 0.001). Table 5 presents a comparison of the parameters between the T2DM patients with sarcopenia and those without sarcopenia (T2DMS-).

**Table 5:** Comparison of the parameters between T2DM patients with and without sarcopenia Data presented as mean ± SD *Significant difference ^#^Independent t-test n: number of patients, BMI: body mass index, ALM: appendicular lean mass, HGS: handgrip strength, ALM/h²: AMMI, AMMI: appendicular muscle mass index, SPPB: Short Physical Performance Battery, SD: standard deviation, HbA1c: hemoglobin A1c, T2DMS+: T2DM patients with sarcopenia, T2DMS-: T2DM patients without sarcopenia

	T2DMS+ (n = 53)	T2DMS- (n = 47)	p-value^#^
Age (mean ± SD)	52 ± 7.61	43.02 ± 7.17	<0.05*
BMI (mean ± SD)	25.2 ± 4.81	26.6 ± 4.95	0.14
Duration of diabetes	8.45 ± 5.45	3.75 ± 1.02	<0.05*
ALM (mean ± SD)	18.5 ± 4.14	21.19 ± 3.95	<0.0014*
ALM/h^2^ (mean ± SD)	7.09 ± 1.34	8.05 ± 0.98	<0.05*
HGS (mean ± SD)	14.97 ± 3.83	25.78 ± 4.34	<0.05*
SPPB score	6.49 ± 1.4	10.06 ± 0.76	<0.05*
HbA1c (mean ± SD)	8.54 ± 1	7.5 ± 1.47	0.00037*

The study compared T2DM patients with sarcopenia (T2DMS+) and those without sarcopenia (T2DMS-). T2DMS+ patients were significantly older (52 ± 7.61 compared with 43.02 ± 7.17 years) and had a longer disease duration (8.45 ± 5.45 compared with 3.75 ± 1.02 years), both with p-values < 0.05. T2DMS+ patients had lower ALM and ALM/h² (p < 0.05), reduced HGS (14.97 ± 3.83 compared with 25.78 ± 4.34, p < 0.05), and lower SPPB scores (6.49 ± 1.4 compared with 10.06 ± 0.76, p < 0.05). Waist circumference and HbA1c levels were also significantly higher in the T2DMS+ group (p < 0.05), indicating a greater metabolic burden and reduced muscle mass and function in diabetic patients with sarcopenia. Other parameters, such as BMI, visceral fat area, and body fat percentage, did not show significant differences. Table 6 shows the proportions of obese and non-obese diabetics with sarcopenia.

**Table 6:** Proportions of obese and non-obese diabetics with sarcopenia ^#^Chi-square test *Significant difference n: number of patients, T2DM: type 2 diabetes mellitus

	T2DM patients with sarcopenia (n = 53)
Obese	34 (64.15%)
Non-obese	19 (34.85%)
p-value^#^	0.0001*

Table 6 shows that sarcopenia was more prevalent in obese diabetics (64.15%, n = 34/53) compared to non-obese diabetics (35.85%, n = 19/53), with an odds ratio of 3.41 (95% CI: 1.38-8.39, p = 0.0001).

## Discussion

This study was initially conceptualized as a cross-sectional observational study focused on diabetic subjects. However, given the limited research on this topic in the Indian context, the study design was expanded to include a comparative analysis. The findings from this study include a detailed comparison between T2DM patients and non-diabetic subjects that highlights significant differences in age, BMI, duration of diabetes, gender distribution, and prevalence of sarcopenia.

Prevalence of sarcopenia and age correlation

This study also revealed that a striking 53% (n = 53/100) of the T2DM patients exhibited sarcopenia, compared with only 17% (n = 17/100) of the non-diabetic patients. The age analysis revealed that 75.47% (n = 40/53) of the T2DM patients with sarcopenia belonged to the 48-60 age group, and this result underscores the age-related sarcopenic trend in diabetics and is consistent with the existing literature, such as the study by Kim et al. of sarcopenia in Korean T2DM patients in which the prevalence of sarcopenia in the patients with diabetes and those in the control group was 15.7% and 6.9%, respectively [10]. Cui et al. observed that the prevalence of sarcopenia in T2DM increased progressively with age; thus, 17.4% in the 65-69 years age group, 28.1% in the 70-74 years age group, 52.4% in the 75-80 years age group, and 60% in the over-80 years age group had sarcopenia [11]. Park et al. likewise found advanced age to be associated with a higher risk of sarcopenia and, conversely, that sarcopenia among non-diabetics was primarily confined to the same age group but occurred at a significantly lower rate [12]. The higher sarcopenia prevalence in T2DM patients is consistent with the understanding that diabetes accelerates muscle deterioration due to impaired insulin signaling, increased inflammation, and oxidative stress. In T2DM, insulin resistance impedes protein synthesis and encourages proteolysis, thereby directly impacting muscle mass and quality [3].

The HbA1c levels in the T2DMG were significantly higher (8.05 ± 1.47%) than in the CG (5.52 ± 0.65%, p < 0.05). This result confirms the poor glycemic control in the diabetic group, which is a known contributor to muscle wasting and sarcopenia. Elevated HbA1c indicates poor glycemic control, which impairs protein synthesis and repair, leading to muscle degradation and increasing sarcopenia risk in diabetic patients [13]. Antidiabetic drugs such as sulfonylureas may increase body fat and reduce skeletal muscle mass, potentially worsening sarcopenia in T2DM patients. Monitoring body composition and glycemic control is essential for comprehensive patient care [14].

Comparison of the prevalence of sarcopenia between diabetic patients with and without sarcopenia

The comparison of T2DM patients with sarcopenia (T2DMS+, n = 53) and those without sarcopenia (T2DMS-, n = 47) revealed some notable distinctions. The mean age of the patients in the T2DMS+ group was 52 ± 7.61 years, significantly higher than the mean age of the T2DMS- group, which was 43.02 ± 7.17 years (p < 0.05). Furthermore, the duration of diabetes was significantly longer in those with sarcopenia (8.45 ± 5.45 years) than in those without (3.75 ± 1.02 years), and this result was also statistically significant (p < 0.05).

Muscle mass and strength were also markedly less in the T2DMS+ group than in the T2DMS- group. Thus, the mean ALM was 18.5 ± 4.14 kg in the sarcopenic patients compared with 21.19 ± 3.95 kg in the non-sarcopenic patients (p < 0.0014). Similarly, the ALM normalized to height squared (ALM/h²), which is a common measure of muscle mass, was significantly lower in the sarcopenic group (7.09 ± 1.34) than in the non-sarcopenic group (8.05 ± 0.98) (p < 0.05).

Functional assessments using HGS and the SPPB also revealed significant differences. HGS was markedly lower in the sarcopenic group (14.97 ± 3.83 kg) compared with the non-sarcopenic group (25.78 ± 4.34 kg), while the SPPB scores averaged 6.49 ± 1.4 and 10.06 ± 0.76, respectively, both with p-values < 0.05. These results indicated impaired muscle function in the sarcopenic diabetic patients. Interestingly, although the mean BMI was slightly lower in the sarcopenic group (25.2 ± 4.81 kg/m²) than in the non-sarcopenic group (26.6 ± 4.95 kg/m²), this difference was not statistically significant (p = 0.14). HbA1c levels, however, were significantly higher in the sarcopenic group (8.54 ± 1.00%) than in the non-sarcopenic group (7.5 ± 1.47%), indicating poorer glycemic control in the sarcopenic individuals (p = 0.00037). Similarly, a study by Volpato et al. reported that a longer duration of diabetes was associated with increased prevalence of sarcopenia [15]. The significantly lower ALM and HGS in the T2DMS+ group indicated compromised muscle function, while the lower SPPB scores highlight the reduced physical performance capacity that is characteristic of advanced sarcopenia [16,17]. Elevated HbA1c in the T2DMS+ group points to a metabolic burden exacerbated by prolonged hyperglycemia that contributed to muscle wasting and physical decline. Consequently, targeted interventions for glycemic control and muscle preservation are essential for diabetic patients at risk of sarcopenia.

Obesity and sarcopenia in T2DM

In our study, 64.15% of T2DM patients with sarcopenia were obese, while 34.85% were non-obese, demonstrating a statistically significant association between obesity and sarcopenia (p = 0.0001). This coexistence of low muscle mass and increased fat mass (termed sarcopenic obesity) is associated with worsened glycemic control, functional decline, and increased cardiometabolic risk. Mechanistically, insulin resistance in T2DM promotes both adiposity and muscle catabolism, while chronic low-grade inflammation (e.g., elevated TNF-α and IL-6) accelerates muscle degradation. Hormonal imbalances, such as reduced testosterone and growth hormone levels, and mitochondrial dysfunction further contribute to muscle loss in obese diabetics. Similarly, Yogesh et al. found sarcopenia (in 60% of patients) and sarcopenic obesity (in 40% of patients) to be common in Indian adults with T2DM [18], although their older cohort (≥60 years) may account for the slightly different prevalence. Similar associations have been described by Stenholm et al. [19] and Mesinovic et al. [2], underscoring that sarcopenic obesity reflects a complex interplay of metabolic, inflammatory, and hormonal pathways rather than obesity alone.

Limitations

The relatively small sample size of 100 per group limits the generalizability of the findings presented here, especially given India’s diverse population. Potential confounding variables such as diet, physical activity, socioeconomic status, and other coexisting conditions were not accounted for, although these factors may influence the prevalence of sarcopenia. Future studies should address these limitations by recruiting larger and more diverse samples and incorporating multivariate analyses to adjust for potential confounders. Collecting detailed lifestyle and comorbidity data would further improve the accuracy of prevalence estimates and clarify the independent effect of T2DM on sarcopenia. Although validated methods such as bioimpedance analysis, HGS, and the SPPB were used, these methods are less accurate than advanced techniques such as dual energy X-ray absorptiometry or magnetic resonance imaging. Additionally, the study did not involve the collection of long-term outcome data relating to the effect of sarcopenia on mortality, disability, or quality of life for T2DM patients. Accordingly, longitudinal research is needed.

## Conclusions

The findings presented here draw attention to the considerable burden of sarcopenia among individuals with T2DM, demonstrating a strong association among reduced muscle mass, lower strength, and impaired physical performance in this population. However, these results should be interpreted in light of the study’s cross-sectional design, which precludes establishing causality, and the potential influence of unmeasured confounding factors such as diet, physical activity, socioeconomic status, and other comorbidities. The observed high prevalence of sarcopenia in T2DM patients is concerning, as the condition may increase physical frailty, diminish functional independence, and complicate efforts to maintain stable metabolic control. These findings thus reinforce the importance of incorporating regular assessments of muscle health into the routine care of individuals with diabetes.

Given the complex relationship between muscle loss and diabetes, with metabolic disturbances potentially contributing to muscle decline, effective management must address multiple factors. Dietary interventions that support muscle maintenance and overall metabolic balance are particularly important. Ensuring adequate protein intake from diverse, nutrient-rich sources and attention to key micronutrients can play a supportive role. Structured meal planning tailored to improving blood sugar levels may also help mitigate the progression of sarcopenia in T2DM patients. Ultimately, addressing sarcopenia in the context of diabetes requires a comprehensive strategy that includes early identification, personalized physical activity programs, and nutritional support. Such an approach can help preserve mobility, improve quality of life, and support positive long-term health outcomes for people living with T2DM.
